# Reply to: Correspondence on “Cardiomyopathy in cirrhosis: From pathophysiology to clinical care”

**DOI:** 10.1016/j.jhepr.2024.101129

**Published:** 2024-06-05

**Authors:** Hongqun Liu, Jwan A. Naser, Grace Lin, Samuel S. Lee

**Affiliations:** 1 Liver Unit, University of Calgary Cumming School of Medicine, Calgary, AB, Canada; 2 Division of Cardiology, Mayo Clinic, Rochester, MN, USA

To the Editor:

We are delighted that our recent review^1^ has stimulated two very thoughtful and thought-provoking responses.^2^^,^^3^ Cailes and colleagues raise two points. First, they propose that a dobutamine challenge stress test, with a cut-off value of >25% increment in cardiac output, be added to the diagnostic criteria for cirrhotic cardiomyopathy (CCM), which currently includes only resting echocardiographic systolic and diastolic parameters. They base this suggestion on their previous study showing that failing to achieve this increment is associated with a four-fold higher risk of developing hepatorenal syndrome.^4^ Their second point questions whether prolonged electrocardiographic QTc interval should be considered as a diagnostic criterion – they think not.

It should be emphasized that neither the 2005 World Congress of Gastroenterology (WCG) nor the 2019 Cirrhotic Cardiomyopathy Consortium (CCC) criteria^5^ were intended to be the definitive final criteria for the diagnosis of CCM. Instead, the intent was that they be interim criteria serving as a basis for discussion and eventual refinement according to further studies. In particular, clinical outcome studies of endpoints such as hepatorenal syndrome and mortality, including the aforementioned hepatorenal syndrome study, will be crucial in revising and refining the criteria. We agree entirely that a cardiovascular stress test of some type is important in making the diagnosis of CCM – this was the subject of intense discussion at the CCC consensus-developing meeting in 2018, but ultimately was not added to the published 2019 criteria because there was no clearly accepted and validated stress test then (Lee SS, Lin G: unpublished observations). So, while Cailes and coworkers’ suggestion to use the dobutamine challenge test is certainly worth considering, we believe that at present it is premature to add this to the 2019 CCC criteria until further validation studies are performed.

The same can be said for their contention that prolonged QTc should not be added to the diagnostic criteria, which is something that we had suggested as a topic for discussion. Although Cailes *et al.* and other groups have reported no relationship between prolonged QTc and post-transplant mortality, this point is still controversial as other centres have found such a correlation with both pre- and post-transplant mortality.^6^ Whether prolonged QTc is a part of a spectrum of other electrophysiologic abnormalities that contribute to the pathogenesis of arrhythmias such as atrial fibrillation^7^ remains unsettled. However, we agree that there is no direct correlation between QTc and cardiac contractility. Therefore, any notion of adding prolonged QTc to the diagnostic criteria is also premature and requires further investigation.

Regarding the comments of Lo Russo and colleagues,^3^ they highlight the complex connections and inter-relationships between liver and heart diseases, and we generally agree with their comments. However, we disagree with one specific comment: “CCM can be considered end-stage cardiomyopathy with multiple causes, including coronary artery disease (CAD)”. The definition of cirrhotic cardiomyopathy specifically excludes known primary cardiac conditions such as CAD. Moreover, CCM rarely manifests with the clinical features of end-stage cardiomyopathy with overt failure, it almost always presents as heart failure with preserved ejection fraction.

Otherwise, these investigators’ points are well taken, especially the need to study the role of cardiac dysfunction as a contributing pathogenic factor or important sequela to acute-on-chronic liver failure.

In summary, we appreciate the interesting comments and important work in the field that both groups are doing. These letters underscore the need to do further research on cirrhotic cardiomyopathy and the broader topic of liver-heart connections.^8^

## Financial support

No financial support was provided for this work.

## Conflict of interest

HL: nil to disclose. JAN: nil to disclose. GL: Research grants from: Pfizer, Biotronik, IONIS, Anumana. Consulting for: Boston Scientific, IONIS. Equity: Empallo, HeartScreen Health. SSL: consulting or speaking for: Abbvie, Gilead, Grifols, Intercept, Jazz Pharmaceuticals, Lupin, Oncoustics.

Please refer to the accompanying ICMJE disclosure forms for further details.

## Authors' contributions

All authors contributed to the discussion and writing of this letter.
